# Increased risk of prediabetes among virally suppressed adults with HIV in Central Kenya detected using glycated haemoglobin and fasting blood glucose

**DOI:** 10.1002/edm2.292

**Published:** 2021-07-17

**Authors:** Anne Njoroge, Orvalho Augusto, Stephanie T. Page, Christine Kigondu, Margaret Oluka, Nancy Puttkammer, Carey Farquhar

**Affiliations:** ^1^ Department of Global Health University of Washington Seattle Washington USA; ^2^ Department of Research and Programs Kenyatta National Hospital Nairobi Kenya; ^3^ Department of Medicine University of Washington Seattle Washington USA; ^4^ Department of Pathology University of Nairobi Nairobi Kenya; ^5^ Department of Pharmacology and Pharmacognosy University of Nairobi Nairobi Kenya; ^6^ Department of Epidemiology University of Washington Seattle Washington USA

**Keywords:** HIV, prediabetes, type 2 diabetes, viral suppression

## Abstract

**Aims:**

As survival among people living with HIV (PLHIV) improves with universal HIV treatment, new strategies are needed to support management of co‐morbidities like type 2 diabetes (T2D). We assessed prediabetes and T2D prevalence and risk factors using haemoglobin A1c (HbA1c) among PLHIV on antiretroviral therapy (ART) in Central Kenya.

**Methods:**

This cross‐sectional study, conducted at a rural and urban site, enrolled PLHIV aged ≥35 years on ART for at least 5 years. HbA1c was assayed using Cobas b 101^®^, a point‐of‐care device. HbA1c levels ≥6.5% were considered diagnostic of T2D. For pre‐diabetic HbA1c levels (5.7%–6.4%), participants were requested to return the following day for a fasting blood glucose (FBG) to rule out T2D. Risk factors were assessed using multivariable log‐binomial regression.

**Results:**

Of the 600 completing study procedures, the prevalence of diabetes was 5% (30/600). Ten participants were known to have diabetes; thus, prevalence of newly diagnosed T2D was 3.4% (20/590). Prevalence of prediabetes (HbA1c 5.7%–6.4%) was 14.2% (84/590). Significant predictors of elevated HbA1c were increase in age (Prevalence ratio [PR]: 1.10, CI: 1.02, 1.18, *p *= .012), hypertension (PR: 1.43, CI: 1.07–2.3, *p *= .015), central adiposity (PR: 2.11, CI: 1.57–2.84, *p *< .001) and use of Efavirenz (PR: 2.09, CI: 1.48, 2.96, *p *< .001).

**Conclusion:**

There is a high prevalence of prediabetes, a significant predictor of T2D, among PLHIV in Central Kenya. Point‐of‐care HbA1c may help identify PLHIV with prediabetes in a single screening visit and provide an opportunity for early intervention.

## Graphical abstract

The article highlights the need for T2D screening especially among African people with HIV who are aviremic using standard diagnostic measures. The results show a high prevalence of prediabetes (14%) with a significant proportion of them expected to develop overt diabetes and ultimately its costly complications.

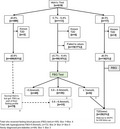

## 1 INTRODUCTION

Globally, people living with HIV (PLHIV) have been shown to have a higher prevalence of type 2 diabetes (T2D) and prediabetes relative to those without HIV.^1^ Chronic systemic inflammation and toxicity related to antiretroviral therapy (ART) have been linked to pancreatic insufficiency and peripheral insulin resistance.^2^, ^3^ With increased life expectancy following early initiation of ART, PLHIV are also experiencing age‐associated metabolic conditions, including T2D and cardiovascular disease.^4^ We sought to characterize the prevalence of prediabetes, T2D and associated risk factors for hyperglycaemia among ART‐experienced PLHIV in a rural and urban environment in Central Kenya.

Prediabetes is important to identify as it has a better predictive value for developing T2D than individual risk factors including obesity and familial history.^5^, ^6^ Outside of Africa, the incidence of T2D among PLHIV is high. In South East Asia, it ranges from 3.4 to 11.1 per 100 person years.^7^, ^8^ However, findings on prevalence of T2D among PLHIV in sub‐Saharan Africa (SSA) are mixed. While earlier studies found up to fourfold increased risk for T2D among PLHIV compared to individuals without HIV,^9^, ^10^, ^11^ a review of recent studies in SSA reported T2D prevalence closer to twofold greater among people with HIV compared to adults without HIV, with prevalence ranging from 0.5%–9.3% among PLHIV and 0.5%–3.6% among individuals without HIV.^12^ These conflicting findings could be due to use of different tests and criteria for diagnosis of T2D. Heterogeneity in ART use, for example including both ART‐naïve and ART‐experienced patients could also explain the mixed findings. Earlier studies also suggested that treatment with protease inhibitors (PI), high viral load and low CD4 count were predictors of T2D among PLHIV.^13^ With universal ART roll‐out, there is a need to identify persistent risk factors for T2D among PLHIV with immune reconstitution hence a high CD4 count and viral suppression and are using newer classes of ART drugs.

Optimal screening approaches for diabetes suitable for PLHIV in resource‐limited settings are critical first steps in this process. Glycated haemoglobin (HbA1c) captures the glycaemic status over a 3‐month period and does not require fasting, therefore a feasible and acceptable screening tool in this population.^14^ While older studies reported that compared to fasting blood glucose (FBG) or oral glucose tolerance test (OGTT), HbA1c underestimated T2D prevalence in PLHIV populations, these studies were conducted among adults with severe HIV‐associated immunosuppression.^15^, ^16^ Recent studies that include PLHIV on ART have found similar test performance comparing HbA1c to other screening tests.^17^, ^18^ Availability of consistent and standardized point‐of‐care (POC) HbA1c devices has increased access to HbA1c testing and POC HbA1c could be a feasible screening tool in the Kenyan population.^19^, ^20^ With this in mind, we conducted a cross‐sectional study using HbA1c screening and a confirmatory blood glucose to assess the burden of prediabetes, T2D and associated risk factors for hyperglycaemia among ART‐experienced PLHIV in Central Kenya.

## 2 SUBJECTS, MATERIALS AND METHODS

### 2.1 Study subjects and setting

During a routine clinic visit, PLHIV aged ≥35 years were recruited from the HIV Comprehensive Care Centres (CCC) at Kiambu and Kerugoya, urban and rural referral hospitals, respectively, through systematic random sampling from January through July 2018. Eligibility criteria included having been on ART for at least 5 years and having had a clinical review within the previous 6 months. Patients with a history of blood transfusion or anaemia in the preceding 6 months and pregnant women were excluded. Written informed consent was obtained by a dedicated study nurse prior to all study procedures. The study was approved by the Kenyatta National Hospital/University of Nairobi Ethics Review Committee and the University of Washington Human Subjects Division.

At enrolment, a questionnaire adapted from the World Health Organization (WHO) Stepwise Approach to Surveillance (STEPS) instrument (Annex 1) was administered by a trained study nurse to obtain demographics, risk factors such as smoking and alcohol use, family history of diabetes and prior diagnosis or treatment of diabetes and/or hypertension. Questions assessing participants’ history of screening for diabetes, including type of screening test used and the indication for screening, and knowledge of increased risk of hyperglycaemia secondary to HIV were asked, since people with HIV are expected to have annual screening for diabetes as per the national HIV treatment guidelines.^21^ Factors influencing haemoglobin levels such as other medications and self‐reported haemoglobinopathies, including sickle cell anaemia, were also obtained.

HIV‐specific data including the date of HIV diagnosis, current and previous ART regimens and blood pressure (BP) readings from the two previous clinic visits were extracted by the study nurse from participants’ clinical charts and confirmed from the electronic medical records. Weight, height, waist and hip circumferences were measured according to WHO international standards.^22^ A blood pressure reading was obtained with the participant seated and legs uncrossed, as part of routine clinic visit procedures.

### 2.2 Glucose screening

The HbA1c assay was done using *Cobas b 101* ^®^ by Roche^©^, a point‐of‐care instrument which measures per cent HbA1c and mmol/mol HbA1c by photometric transmission measurement. Internal quality controls were implemented as per the device manufacturer's instructions and run every 2 weeks. The device's coefficient of variation provided by the manufacturer was 0.19% HbA1c.^23^ Venous samples were obtained from 1% of the participants and assayed in an accredited laboratory for comparison with the POC values, showing good collinearity, with a correlation coefficient of 0.97 and a % mean difference of 2%.

**FIGURE 1:**
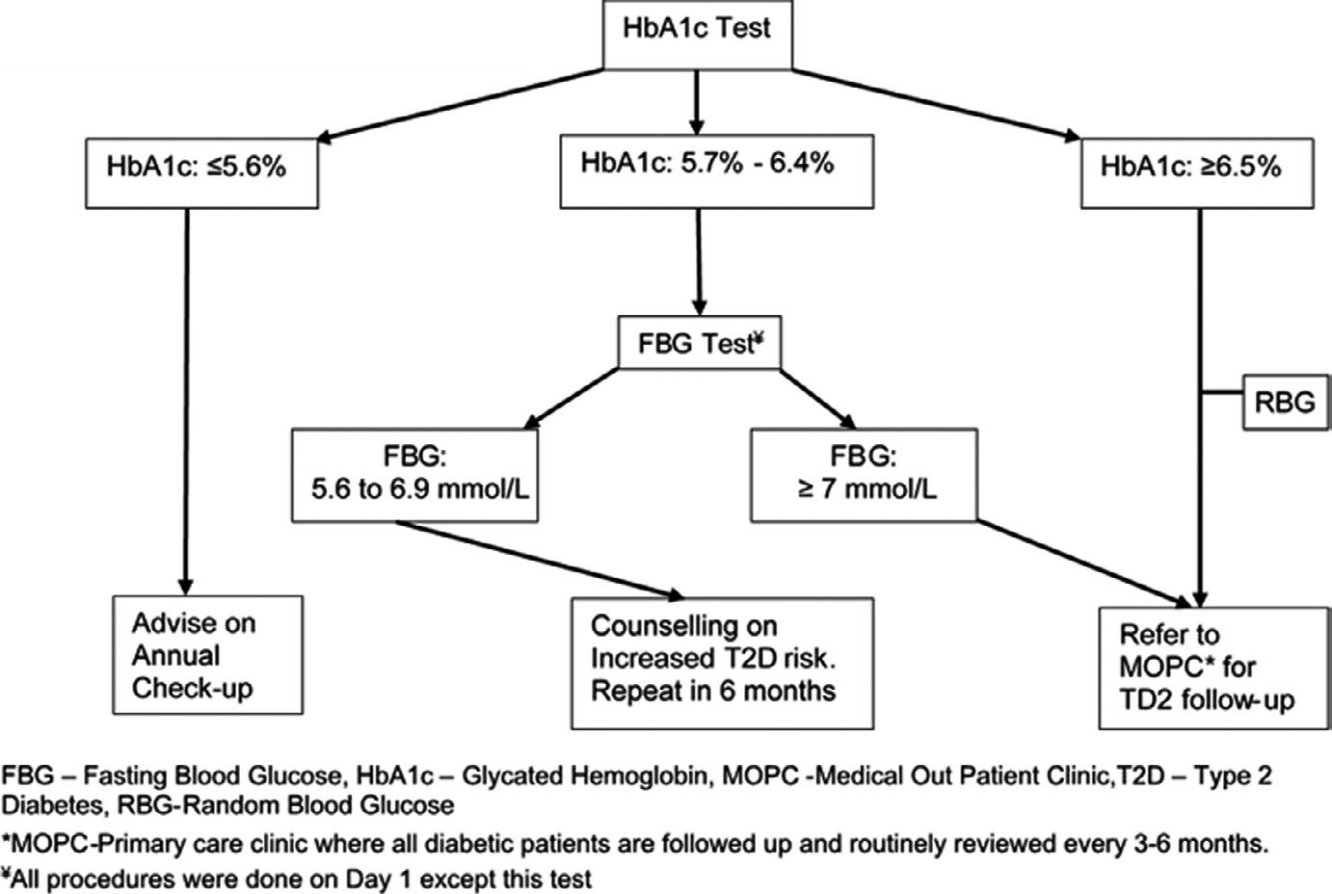
Study procedures flowchart

For HbA1c levels ≥6.5% (≥48 mmol/mol), a random blood glucose obtained on the same day was used to confirm T2D diagnosis [Figure 1], as per the American Diabetes Association (ADA) guidelines where a diagnosis needs to be confirmed using a different test. For HbA1c levels between 5.7% and 6.4% (39–47 mmol/mol), participants were requested to return while fasting the following day for a fasting blood glucose using *Accu‐Check^®^* by Roche©. Some patients who also needed a fasting lipid profile (details not presented here) also got a fasting blood glucose regardless of their HbA1c.

### 2.3 Definitions of diabetes and hypertension

Hypertension was defined by two parameters: (1) two readings where systolic BP ≥140 mm Hg and/or diastolic BP ≥90 mm Hg,^24^ using the enrolment BP measurement and a second BP reading from the clinical chart or (2) reported use of antihypertensive medications. Prediabetes was defined as a HbA1c between 5.7 and 6.4% (39–47 mmol/mol). Diabetes was defined by three parameters: (1) a HbA1c ≥6.5% and random blood glucose ≥11.1 mmol/L or (2) a HbA1c between 5.7 and 6.4% and fasting blood glucose ≥7.0 mmol/L or (3) reported use of insulin or oral hypoglycaemics.^25^, ^26^

### 2.4 Statistical analysis

The sample size was based on an a priori estimated prevalence of diabetes among the PLHIV population of 16%–25%.^11^, ^27^ Using a normal approximation to the binomial distribution, we needed a sample size of 588 to observe an estimate of 25% prevalence of T2D with a precision interval of ±3.5%.

Due to lower‐than‐expected numbers of participants with diabetes in the sample, prediabetes and diabetes were combined to create a binary outcome for the regression analysis. Multivariable log‐binomial regression was used to assess factors associated with prediabetes and diabetes. Covariates considered include age, sex, family history of T2D, time since HIV diagnosis, duration on ART, individual ART drugs, alcohol use, hypertension, body mass index (BMI) and waist circumference. Following bivariable analysis, covariates with a *p*‐value of ≤.05 were included in the multivariable model.

## 3 RESULTS

### 3.1 Cohort demographics and prevalence of prediabetes and T2D

Of the 600 participants who completed all study procedures, 383 (63.8%) were female. [Table 1] The median age was 46.8 years (interquartile range [IQR]: 41.6, 53.1).

**TABLE 1:** Demographics, anthropometric measurements and HIV‐related characteristics among PLHIV in Central Kenya

Covariate	All participants	Urban Clinic	Rural Clinic
	*n* = 600	*n* = 302	*n* = 298
	Median (IQR) or *n* (%)	Median (IQR) or *n* (%)	Median (IQR) or *n* (%)
Age (years)	46.8 (41.6, 53.1)	46.6 (41.2, 53.0)	46.9 (42.0, 53.4)
Sex (female)	383 (63.8%)	177 (58.6%)	206 (69.1%)
Education Level			
Primary	334 (55.7%)	155 (51.3%)	179 (60.1%)
Secondary	191 (31.7%)	107 (35.4%)	84 (28.2%)
Post‐Secondary	56 (9.4%)	34 (11.3%)	22 (7.3%)
None	19 (3.2%)	6 (2.0%)	13 (4.4%)
Time since HIV diagnosis (years)	9.3 (7.6, 11.1)	9.1 (7.7, 11.0)	9.7 (7.6, 11.4)
Time on ART (years)	8.1 (6.5, 10.0)	8.0 (6.7, 9.6)	8.3 (6.4, 0.3)
Viral load (>0 copies/ml)	45 (7.5%)	10 (3.6%)	35 (12.2%)
Current CD4 (cells/ml)	512 (354, 690)	482 (324, 660)	540 (387, 719)
Current ART Regimen			
2NRTI+NNRTI	560 (93.3%)	284 (94.0%)	276 (92.6%)
NRTI+NNRTI+PI	39 (6.5%)	17 (5.6%)	22 (7.4%)
Other	1 (0.2%)	1 (0.4%)	0 (0.0%)
Smoking			
Never	431 (71.8%)	206 (68.2%)	225 (75.5%)
Previous	144 (24.0%)	82 (27.2%)	62 (20.8%)
Current	25 (4.2%)	14 (4.6%)	11 (3.7%)
Alcohol Use¥	63 (10.5%)	42 (13.9%)	21 (7.0%)
Hypertension¥	215 (35.8%)	79 (26.2%)	136 (45.6%)
Waist‐Hip Ratio (≥0.9 for males and ≥0.85 for females)	329 (54.8%)	158 (52.3%)	171 (57.4%)
Waist Circumference (>102 for males and >88 cm for females)	200 (33.3%)	102 (33.8%)	98 (32.9%)
Previously diagnosed T2D	10 (1.7%)	3 (1.0%)	7 (2.3%)
Family history of T2D	125 (20.8%)	60 (19.9%)	65 (21.8%)
Previous Blood Sugar Screening¥	145 (24.2%)	56 (18.5%)	89 (29.9%)
Random Blood Glucose	136 (93.8%)	54 (96%)	82 (92%)
Fasting Blood Glucose	8 (5.5%)	1 (2%)	7 (8%)
Haemoglobin A1c	1 (0.7%)	1 (2%)	0 (0%)
Reasons for Blood Sugar Screening¥			
Free, for example medical camp	52 (35.9%)	19 (34%)	33 (37%)
Test annually by myself	41 (28.3%)	12 (21%)	29 (33%)
Test annually by physician	27 (18.6%)	13 (23%)	14 (16%)
Part of clinical work‐up	22 (15.1%)	9 (16%)	13 (15%)
Due to familial history	1 (0.7%)	1 (2%)	0 (0%)
Others	2 (1.4%)	2 (4%)	0 (0%)

Abbreviations: 3TC, lamivudine; ART, antiretroviral therapy; BMI body mass index; IQR, interquartile range; NNRTI, non‐nucleoside reverse transcriptase inhibitor; NRTI, nucleoside reverse transcriptase inhibitor; PI, protease inhibitor; T2D, type 2 diabetes.

¥*p*‐value <.05 comparing urban to rural clinic.

The median time since HIV diagnosis was 9.3 years (IQR: 7.6, 11.1), while the median duration on ART was 8.1 years (IQR: 6.5, 10.0). Median current CD4 was 512 cells/ml (IQR: 354,690) and though 45 individuals (7.5%) had a detectable viral load, only 22 (3.7%) had more than 1000 copies/ml, the cut‐off for virologic treatment failure. A majority (93%) were on a combination of two nucleoside reverse transcriptase inhibitors (NRTI); tenofovir [TDF] or zidovudine [AZT], lamivudine^28^), and a non‐nucleoside reverse transcriptase inhibitor (NNRTI) efavirenz^28^ or nevirapine. Only 7% were using a protease inhibitor. One hundred and thirty‐six (45.6%) participants from the rural clinic were hypertensive compared to 79 (26.2%) from the urban clinic (*p*‐value: <.001). Of note, among hypertensive patients on antihypertensives, none of their medication information was recorded in the electronic medical system.

**FIGURE 2:**
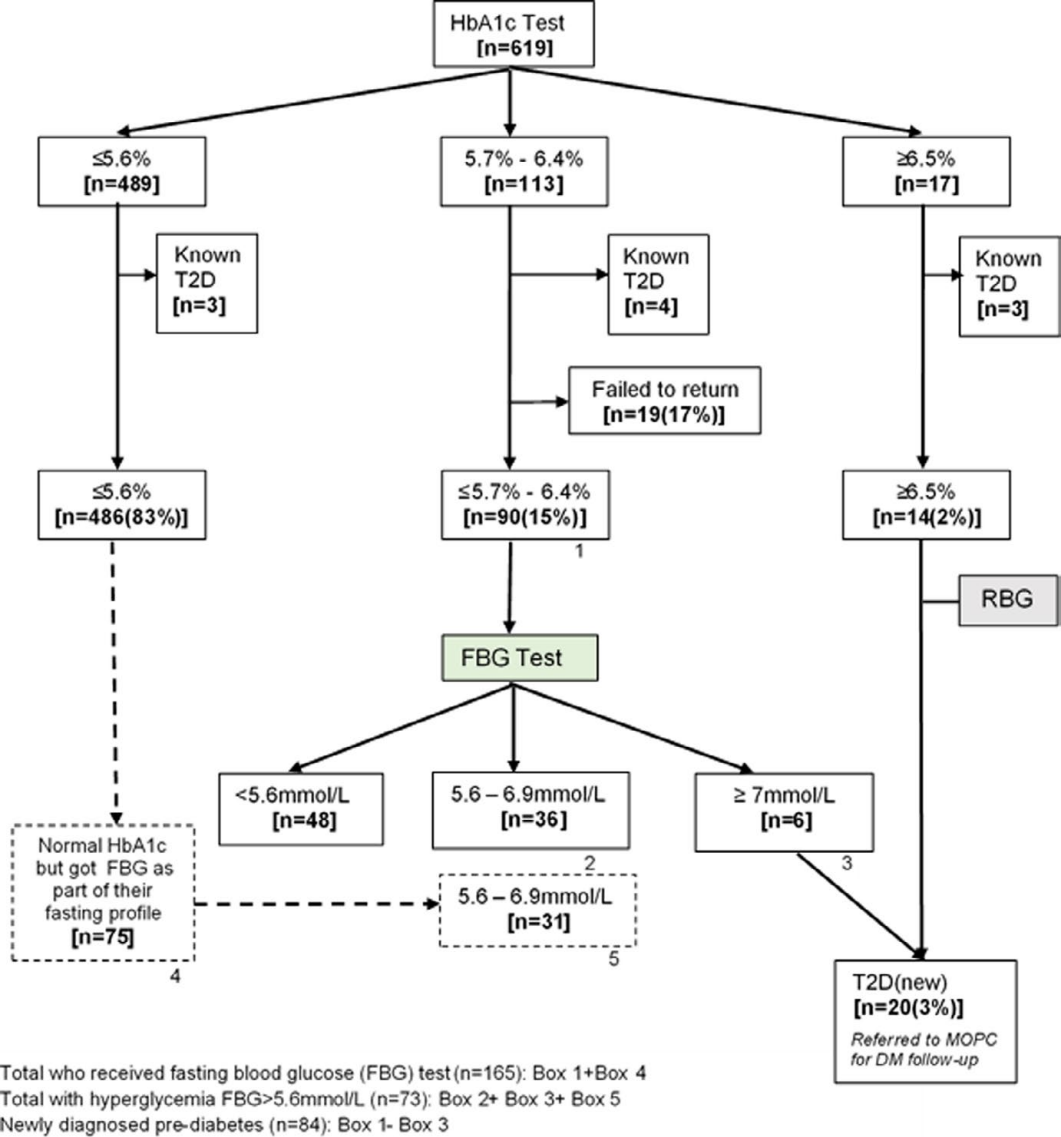
Prediabetes and diabetes testing and results among 619 HIV‐positive study participants

At enrolment, 10 (1.7%) of 600 participants were known to have diabetes. Among those not known to have diabetes, 20 were newly diagnosed to have T2D. The overall prevalence of diabetes was therefore 5% (30/600) while the prevalence of newly diagnosed T2D was 3.4% (20/590), with fourteen cases (2.4%) having diabetes as defined by HbA1c ≥6.5% (≥48 mmol/mol) and random blood glucose >11.1 mmol/L and six cases having diabetes as defined by HbA1c 5.7% ‐ 6.4% and fasting glucose ≥7.0 mmol/L [Figure 2]. Of the participants requested to return for fasting glucose to rule out diabetes, 17% (19/113) failed to return. Excluding participants known to have T2D, the prevalence of newly diagnosed prediabetes (HbA1c 5.7%–6.4%) was therefore 14.2% ([84/590) [Figure 2]. Overall, newly diagnosed hyperglycaemia (T2D and prediabetes) was 21% (62/299) in Kiambu compared to 14% (42/291) in Kerugoya. (*p *= .045).

### 3.2 **Factors associated with prediabetes and diabetes**.

Factors associated with a diagnosis of prediabetes or diabetes are shown in Table 2 and included age, hypertension, central obesity and an ART regimen containing EFV. In multivariable log‐binomial regression analysis, age was significantly associated with prediabetes and diabetes, with a 13% increase in the prevalence ratio for every 5‐year increase in age (adjusted prevalence ratio [aPR]: 1.13, CI: 1.03, 1.25, *p *= .004). Hypertensive patients were 1.61 times as likely to have prediabetes and diabetes compared to those without hypertension (aPR: 1.61, CI: 1.15, 2.26, *p *= .006) [Table 2]. Hyperglycaemic HbA1c values were twice as likely among those with central adiposity (waist circumference>102 cm for males and >88 cm for females) compared to those with normal waist circumference (aPR: 2.09, CI: 1.50, 2.92, *p* < .001). BMI and waist circumference were not included in the same model as these variables were highly colinear (Pearson's *r* = 0.83).

**TABLE 2:** Predictors of diabetes and prediabetes among 590 persons living with HIV

Covariate	Unadjusted PR (95% CI)	*p*‐value for unadjusted PR	Adjusted PR^1^ (95% CI)	*p*‐value for adjusted PR
Age (5 years)	1.16 (1.05, 1.27)	.**002**	1.13 (1.03, 1.25)	.**004**
Sex (male)	0.71 (0.48, 1.05)	.08		
Time since HIV diagnosis (years)	1.05 (0.98, 1.14)	.22		
Time on ART	1.06 (0.98, 1.16)	.14		
Family history of DM	1.42 (0.96, 2.09)	.08		
Alcohol Use	0.70 (0.36, 1.37)	.29		
Hypertension	1.69 (1.19, 2.38)	.**003**	1.61 (1.15, 2.26)	.**006**
Waist Circumference (>102 for males & >88 cm for females)	2.19 (1.55, 3.09)	**<.001**	2.09 (1.50, 2.92)	**<.001**
Viral load (detectable)	0.73 (0.34, 1.58)	.425		
Current CD4(cells/ml)	1.00 (0.99, 1.01)	.087		
ART (Having the drug as part of their current or previous combination)				
TDF (*n* = 456)	1.62 (1.00, 2.63)	.05		
NVP (*n* = 459)	1.08 (0.71, 1.64)	.74		
EFV (*n* = 274)	1.83 (1.28, 2.62)	.**001**	2.09 (1.48, 2.96)	**<.001**
AZT (*n* = 215)	0.77 (0.52, 1.13)	.18		
ABC (*n* = 6)	0.95 (0.16, 5.70)	.95		
LPV/r (*n* = 26)	0.64 (0.22, 1.90)	.43		
ATV/r (*n* = 15)	0.75 (0.19, 2.63)	.67		

Note

Bold values indicates the statistical significance.

Covariates with a *p*‐value of <.05 in bivariable analysis were included in the multivariable model.

Abbreviation: PR, Prevalence Ratio.

People on current or previous regimens containing EFV were twice as likely to have prediabetes and diabetes (aPR: 2.09, CI: 1.48, 2.96, *p *< 0.001) [Table 2]. Time since HIV diagnosis, duration on ART, sex, CD4 count, viral load or being on a PI‐containing regimen were not associated with prediabetes and diabetes in bivariable analyses and were excluded from the multivariable model.

Among the 486 participants who had a normal HbA1c, 75 of them returned the following day for a fasting lipid profile and also got a fasting blood glucose; hence, a total of 165 people had fasting blood glucose [Figure 2]. Using FBG as the outcome, the prevalence of hyperglycaemia (>5.6 mmol/L) was 44% (73/165) and the direction of association between the risk factors and elevated FBG remained the same, although statistical significance was lost due to the smaller sample size.

### 3.3 Diabetes awareness and screening

Overall, 145 participants (24.2%) reported being previously screened for diabetes, with 89 (29.9%) from the rural clinic compared to 56 (18.5%) in the urban clinic, a difference that was statistically significant (*p *= .001). This screening had been done using a random blood glucose (93.8%) or fasting blood glucose (5.5%). Majority of patients (35.9%) reported prior screening because the blood glucose test had been offered free of charge. While self‐initiated blood sugar screening was higher in the rural (70%) compared to the urban clinic (55%), provider‐driven screening (e.g., as part of clinical work‐up) was higher (39%) in the urban clinic compared to 31% in the rural clinic (*p *= .039).

## 4 DISCUSSION

After excluding those with known T2D, overall prevalence of newly diagnosed diabetes using HbA1c was low (3.4%) in this virally suppressed PLHIV cohort. However, prevalence of prediabetes was high at 14.2%. With up to 10% of people with prediabetes progressing to develop diabetes each year, identifying and addressing prediabetes through diet, lifestyle modification or metformin could reduce future risk of diseases associated with T2D such as stroke, myocardial infarction and peripheral arterial disease.^29^ Risk factors associated with prediabetes and diabetes included age, presence of hypertension, abnormal waist circumference and current or prior use of efavirenz. Hypertension was prevalent in over a third (36%) of the population, with higher proportions observed in the rural compared to the urban clinic (46% vs 26%). These results support previously published findings that PLHIV share similar risk factors for diabetes with the general population. Many studies have described an association between diabetes and age, hypertension and central adiposity, in both general populations and PLHIV sub‐populations.^12^, ^30^

Our findings are also consistent with several recent studies which have reported a wide range of values for the prevalence of diabetes and its risk factors among individuals on ART in SSA: diabetes (0.5%–8%),^31^ prediabetes (12%–21%)^11^, ^13^, ^32^, ^33^ and hypertension (12%–39%).^34^, ^35^ Prevalence of diabetes (3.4%) in our study population was similar to these studies and to other recent studies in Kenya among PLHIV which found 2.1%–4.8%.^36^, ^37^ It was also consistent with the overall range of T2D prevalence estimates in Kenya among all adults, regardless of HIV status, at 3.5% to 5%.^38^ Our estimates of T2D among PLHIV in Kenya were lower than estimates in low‐income countries in South East Asia, where estimates of up to 9% have been reported.^7^ These differences could be due to lower prevalence of other risk factors, like alcohol and smoking, in our study population relative to PLHIV in these regions or to other environmental or genetic differences.^30^, ^39^

The association of hyperglycaemia with Efavirenz is also consistent with what has previously been reported in the literature.^9^, ^11^ This is particularly significant in Kenya, where a greater proportion of patients are on TDF and EFV as their first‐line regimen, yet diabetes screening is emphasized only among the small proportion on PI‐containing regimens. Dolutegravir, an integrase inhibitor which has been associated with weight gain and hyperglycaemia,^40^, ^41^ is being rolled out nationally to replace EFV as part of the first‐line regimen. Should the hyperglycaemic effect of both drugs be cumulative, a case can be made for diabetes and prediabetes screening to be offered to all PLHIV as part of comprehensive health services.

Despite being in the ART treatment guidelines, awareness on the need for screening was generally low (24.2%). With the move towards holistic patient care, PLHIV need to be sensitized on the increased risk of prediabetes and diabetes and therefore the need for routine screening. Since self‐initiated blood sugar screening occurred largely when the test was free, cost and affordability should be a key factor when considering feasible diabetes screening strategies.

This study is the first to our knowledge that reports on prevalence of diabetes using the standard ADA HbA1c definition in East Africa. Many previous studies have relied on a single test. A strength of this study is use of a confirmatory test as per the ADA guidelines. Nearly one in five of those asked to return the following day while fasting failed to come back, highlighting the difficulties posed by screening approaches that require multiple clinic visits. Of note, approximately half of those individuals identified as having prediabetes defined as Hgb A1c 5.7–6.4 had normal fasting blood glucose. Ideally, such individuals would undergo OGTT to determine their risk status. However, given the preponderance of other risk factors for cardiovascular disease in this population, their participation in targeted risk reduction strategies such as diet and lifestyle modification or metformin is warranted. With similar prevalence findings to other studies that used fasting glucose, our findings add to the body of literature supporting use of point‐of‐care HbA1c screening, reducing the requirement for a return visit while fasting.^14^, ^20^ This is important because despite a low prevalence of overt diabetes, the marked prevalence of prediabetes and other risk factors among a population who attend clinic regularly highlights an opportunity to screen for these modifiable risk factors.

Our study limitations include the failure to use 75g‐OGTT test, the gold standard, as part of our algorithm for T2D diagnosis. We did not assess the mean corpuscular volume (MCV) in this population, who are likely to have macrocytosis, a factor that could interfere with HbA1c values. We relied on the last haemoglobin (Hb) assessed in the preceding 6 months and did not assess a current Hb to rule out anaemia. Both macrocytosis and current low haemoglobin would likely be associated with falsely low HbA1c levels. Our findings may therefore underestimate the prevalence of elevated HbA1c in this population.

## 5 CONCLUSION

In summary, we believe our findings add support to calls for universal HbA1c screening using POC screening methods as part of comprehensive HIV care services. Our cohort reflects the growing population of older PLHIV who have been on ART for long, have high CD4 counts and have achieved viral suppression. With time and without intervention, it is expected that a significant proportion of those with prediabetes will develop overt diabetes and ultimately its costly complications. In addition to counselling regarding positive lifestyle changes for these PLHIV, longitudinal follow‐up of PLHIV with prediabetes will be important in understanding the risk of progression to T2D and to better inform screening and intervention strategies for diabetes among PLHIV in sub‐Saharan Africa.

## CONFLICT OF INTEREST

The authors have no conflict of interest to declare.

## AUTHORS CONTRIBUTIONS

AN, SP and CF designed the study. AN, CK and MO were significantly involved in implementation of the study and made significant content contributions. AN and CF took lead in interpretation of the results and drafting of the manuscript. OA and NP provided substantial contributions to statistical analysis, interpretation of the results and drafting of the manuscript. All authors critically reviewed and approved the final version.

## Data Availability

The data that support the findings of this study are available on request from the corresponding author. The data are not publicly available due to privacy or ethical restrictions.
